# How do we improve peer review for manuscripts from culturally divergent origins?

**DOI:** 10.12688/f1000research.5704.1

**Published:** 2015-02-10

**Authors:** Anthony Dart

**Affiliations:** 1 Department of Cardiovascular Medicine, BakerIDI Heart and Diabetes Research Institute and Alfred Hospital, Prahran, Australia

**Keywords:** Peer review, cultural, evaluation

## Abstract

Objective and informed peer review is critically important to the progress of science. These standards can sometimes be at risk in the evaluation of manuscripts from less culturally familiar places and such evaluations therefore require particular vigilance. Alternative publication strategies may be particularly helpful in these circumstances.

## Editorial

The progress of clinical science is critically dependent on the selection of material for publication by journals. Although many factors contribute to the editorial decision to accept or reject any individual submission, the results of peer review are in most cases of paramount importance. It is therefore essential that the process of peer review be conducted in an objective and unbiased way. Most reviewers recognise the importance of their task and strive to give such an opinion. The task becomes even harder when evaluating submissions from different cultures and can even lead to the perception of bias. My involvement in clinical science originating from mainland China has alerted me to the obstacles that arise in the evaluation of work from less familiar, at least to western reviewers, cultures.

Some examples: Disbelief has been expressed on data showing a zero prevalence of cigarette smoking amongst pregnant women ignoring the fact that cigarette smoking is rarely practised by Chinese women, whether pregnant or not; similarly disbelief at the very low prevalence of coffee drinking amongst this cohort ignores the fact that, outside of the major cities, coffee drinking is uncommon. Perhaps more worryingly, a reviewer of a study in children involving blood sampling commented that the study was unethical and would not have been permitted in western countries, despite the fact that several similar studies had indeed been performed in Europe and North America and that parents and children had both given consent. More than one has found the English to be of insufficient standard to permit publication. As an example a recent reviewer commented “Overall, there were many places where the language and grammar need to be improved”. I make no claim as a literary stylist, but I do think my written English is adequate and I wrote the manuscript under consideration in its entirety. More importantly, I rarely, if ever, receive this remark in relation to manuscripts emanating from Australia. Disappointingly these comments have sometimes been proffered in a derogatory and demeaning manner, again something I have not experienced in relation to manuscripts clearly emanating from Australia.

Is there a solution? Some journals attempt to achieve an unbiased evaluation by not revealing authors names or affiliations when requesting peer review and this is sometimes able to adequately conceal the origin of the work. However in many cases it is simply not possible to do this as a result of content, reference to previous work etc. In addition, assessment of the credibility of submitted work may, indeed, require knowledge of the author(s) previous publications. In the traditional publishing paradigm perhaps the only hope lies with journal editors. They need to be vigilant to make sure inappropriate assessment and comments do not contribute to the final decisions and, more importantly, to ensure that reviewers who breach the requirements of impartiality and objectivity are not requested to provide reviews again. As a further step, journals may consider routinely asking the corresponding authors of manuscripts to indicate whether they believe their submissions may have been subjected to a biased assessment based on the origin of the work, if so in what way, and to investigate any such substantive accusations that emerge. An alternative approach, adopted by *F1000Research*, is to publish the paper (and data) in advance of peer review and then publish the (named) reviewers’ comments when these are available. Such an approach should encourage reviewers to be as objective and informed as possible and is likely to curb any tendency to indulge in disparaging or inappropriate comments. Time will tell whether this radical approach, or perhaps a hybrid model whereby reviewers’ comments are more widely available, will remove bias without sacrificing scientific rigour.

